# Home-based palliative approach for people with severe multiple sclerosis and their carers: study protocol for a randomized controlled trial

**DOI:** 10.1186/s13063-015-0695-0

**Published:** 2015-04-23

**Authors:** Alessandra Solari, Andrea Giordano, Maria Grazia Grasso, Paolo Confalonieri, Francesco Patti, Alessandra Lugaresi, Lucia Palmisano, Roberta Amadeo, Giovanni Martino, Michela Ponzio, Giuseppe Casale, Claudia Borreani, Renzo Causarano, Simone Veronese, Paola Zaratin, Mario Alberto Battaglia, on behalf of the PeNSAMI project

**Affiliations:** Unit of Neuroepidemiology, Foundation IRCCS Neurological Institute C. Besta, Via Celoria 11, 20133 Milan, Italy; Multiple Sclerosis Unit, Foundation IRCCS S. Lucia Rehabilitation Hospital, Via Ardeatina 306, 00179 Rome, Italy; Unit of Neuroimmunology, Foundation IRCCS Neurological Institute C. Besta, Via Celoria 11, 20133 Milan, Italy; Department of Medical and Surgical Sciences and Advanced Technologies, University of Catania; MS Center, Neurology Clinic, University Hospital Policlinico Vittorio Emanuele, Via Santa Sofia 78, 95123 Catania, Italy; Department of Neuroscience, Imaging and Clinical Sciences, G. d’Annunzio University of Chieti-Pescara, Via dei Vestini 31, 66100 Chieti, Italy; Department of Therapeutic Research and Medicine Evaluation, Istituto Superiore di Sanità, Viale Regina Elena 299, 00161 Rome, Italy; Associazione Italiana Sclerosi Multipla, Via Operai 40, 16149 Genoa, Italy; Fondazione Italiana Sclerosi Multipla, Via Operai 40, 16149 Genoa, Italy; Antea Charitable Association, Piazza Santa Maria della Pietà 5, 00135 Rome, Italy; Unit of Clinical Psychology, Foundation IRCCS Istituto Nazionale per la Cura dei Tumori, Via Venezian 1, 20133 Milan, Italy; Unit of Palliative Care-Hospice, Niguarda Ca’ Granda Hospital, Piazza Ospedale Maggiore 3, 20162 Milan, Italy; FARO Charitable Foundation, Via Morgari 12, 10125 Turin, Italy

**Keywords:** Multiple sclerosis, Palliative care, Complex intervention, Randomized controlled trial, Qualitative research

## Abstract

**Background:**

Preliminary evidence suggests that palliative care may be useful for people with severe multiple sclerosis (MS). The aim of this study is to determine the effectiveness of a home-based palliative approach (HPA) for people with severe MS and their carers.

**Methods/design:**

This is a single-blind randomized controlled trial with a nested qualitative study. Seventy-five severe MS-carer dyads are being randomized (at three centers, one in each area of Italy) to HPA or usual care (UC) in a 2:1 ratio. Each center has a specially trained team consisting of four professionals (physician, nurse, psychologist, social worker). The team makes a comprehensive assessment of the needs of the dyads. HPA content is then agreed on, discussed with the patient’s caring physician, and delivered over six months. The intervention is not intended to replace existing services. At later visits, the team checks the HPA delivery and reviews/modifies it as necessary.

HPA and UC dyads are assessed at home by a blind examiner at baseline, and three and six months later; they also receive monthly telephone interviews. Dyads assigned to UC receive the examiner’s visits and telephone interviews, but not the team visits.

Primary outcome measures are changes in symptoms (Palliative care Outcome Scale-Symptoms-MS, POS-S-MS), and quality of life (the Schedule for the Evaluation of Individual Quality of Life-Direct Weighting (SEIQoL-DW), not assessed in patients with severe cognitive compromise) at three and six months. Other outcomes are changes in patient functional status and mood; changes in carer quality of life, mood and caregiving burden; costs; incorporation with standard care; unplanned hospital admissions; referrals to hospice; and deaths.

The experience of participants will be evaluated qualitatively by individual semi-structured interviews (HPA patients and carers) and focus group meetings (HPA patients’ caring physicians).

**Discussion:**

The results of our study will show whether the HPA is feasible and beneficial to people with severe MS and their carers living in the three Italian geographic areas. The nested qualitative study will add to the understanding of the strengths and limitations of the intervention.

**Trial registration:**

The trial was registered with Current Controlled Trials (identifier: ISRCTN73082124) on 19 June 2014.

## Background

Multiple sclerosis (MS) is the most common disabling neurological condition of young adults in western countries. It affects over 2.5 million people worldwide, and evidence suggests that incidence is increasing [1]. Around 15% of MS sufferers have a progressive course from the outset (primary progressive MS), and a further 65% develop progressive disease after a variable period with relapsing-remitting disease (secondary progressive MS) [2]. For those with primarily or secondarily progressive MS, treatment options to delay or prevent further clinical worsening are limited. Reduced mobility and compromised sphincter control are among the commonest symptoms, but cognitive impairment, swallowing or speech impairment, pain and sensory disturbances may also be prominent [3-5]. Patients with severe MS are at risk of death from aspiration pneumonia, urinary tract infections, complications of falls and fractures, and sepsis secondary to pressure ulcers. Nevertheless, some highly disabled patients live for many years, although most die in hospital rather than at home [6].

Although robust evidence supporting treatment decisions in advanced MS is lacking, recent guidelines suggest shifting to a palliative approach as the disease worsens [7]. Palliative care, with its focus on the physical, psychological, spiritual and social needs of patients and families, and their active involvement in medical decisions, aims to improve the quality of care and reduce the use of emergency and acute care services. However, palliative care has traditionally been delivered in oncology, and is little used in MS and other neurological diseases. There are issues that are specific to MS: for example, pain due to spasticity requires a different approach to cancer pain management, and cognitive and communication compromise may hamper ability to reveal experiences and express choices. Furthermore, it is difficult to anticipate life expectancy in people with severe MS, who may sometimes need palliative care over an extended period.

It is noteworthy that recent studies on adults with severe MS and their carers [8-11], and also newly diagnosed persons with MS [12,13], indicated similar areas of concern comprising insufficient time spent with health professionals, lack of information exchange, and discontinuity of care.

The current trend of managing chronic and terminal conditions in the community entails the need to implement effective home care models. Informal carers are an essential source of support for MS patients, and in advanced disease informal carers take responsibility for meeting most of their patient’s needs, thereby preventing patient institutionalization. However, the caregiving burden can lead to a decline in carers’ health-related quality of life (HRQOL) and adversely affect their employment and finances.

Evidence supporting a benefit of palliative care is sparse, with most studies assessing needs instead of interventions, or having methodological flaws [14]. We found two randomized controlled trials (RCTs) on the efficacy of palliative care in people with severe MS [8,15]. The UK trial showed that a home-based palliative care service improved symptoms management, reduced caregiver burden and the use of primary and acute hospital services over the short term [8]. The Italian trial (Ne-Pal) included people with severe MS, Parkinson’s disease and related disorders, and amyotrophic lateral sclerosis; the home-based palliative care intervention improved patient HRQOL and some symptoms [15]. Nevertheless, it may not be straightforward to transfer interventions of this sort to different contexts and health systems [16,17].

### Objectives

The primary aim of the present study is to determine the effectiveness of a home-based palliative approach (HPA) on MS symptoms and HRQOL of people with severe MS. Secondary outcomes are changes in patient functional status and mood; changes in carer HRQOL, mood and caregiving burden; costs; incorporation with standard care; unplanned hospital admissions; referrals to hospice; and deaths over the six-month intervention.

## Methods

### Ethical approval and trial registration

The study was given ethical approval by the Foundation IRCCS Neurological Institute ‘C. Besta’ (Besta internal reference numbers 6, 11), the Foundation ‘S. Lucia’ Hospital (internal reference number CE/OSS.27), and the University Hospital of Catania (internal reference number 18/2014/PO) Ethics Committees. The trial is registered with Controlled Clinical Trials (trial registration number ISRCTN73082124).

### Trial design

**Figure 1:**
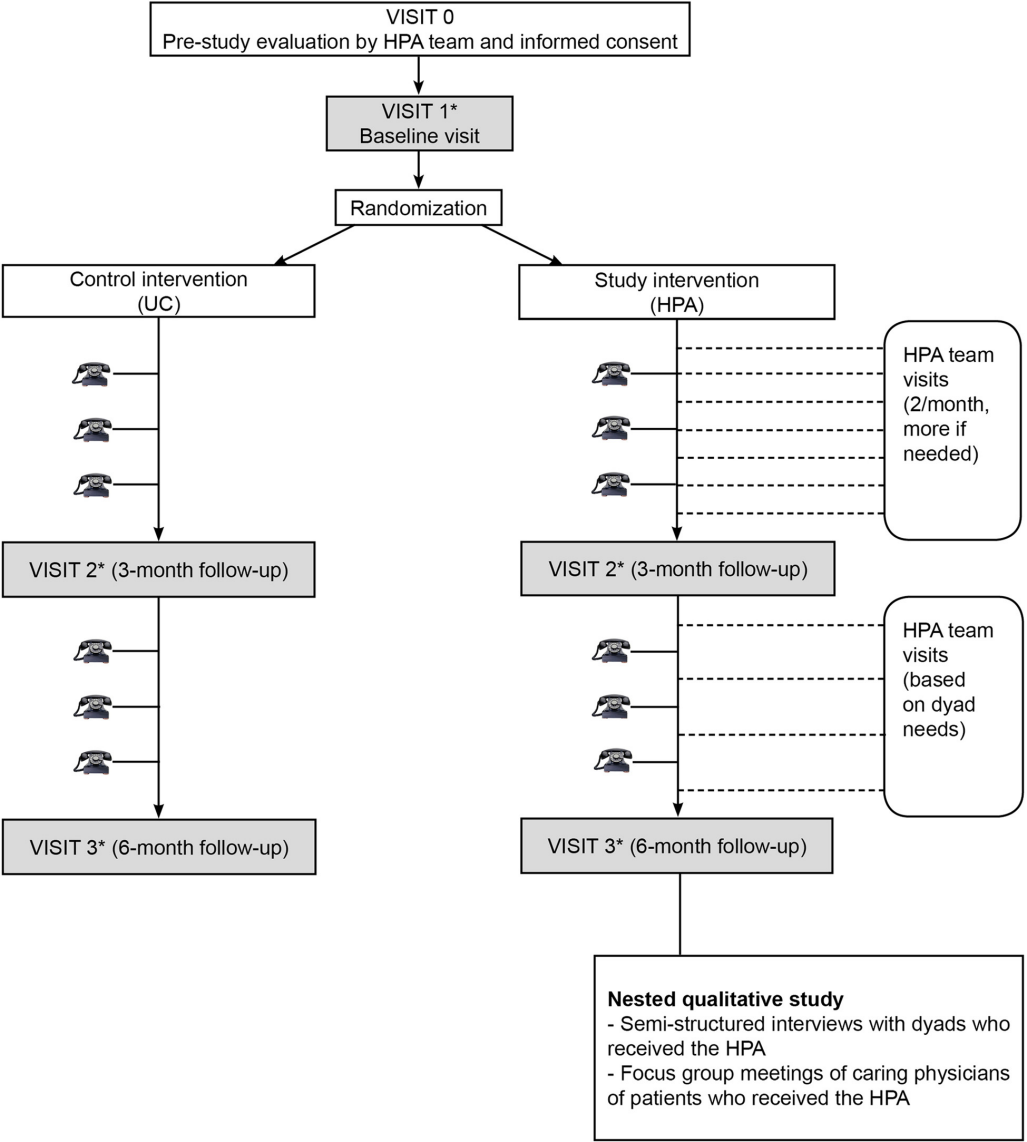
The PeNSAMI trial flowchart. ^*^Visits 1 to 3 performed by a blind examiner;  phone interviews performed by a trained interviewer. HPA, home-based palliative approach; UC, usual care.

This is a multicenter phase II/III single-blind randomized controlled trial. Participants (dyads of adults with severe MS and their carers) are randomized to either the HPA intervention group or usual care (UC). Figure 1 presents the PeNSAMI trial flowchart.

#### Inclusion and exclusion criteria

All adults (18 years or older) who fulfill all the following criteria are potentially eligible:

1. Diagnosis of MS [18]
2. Expanded Disability Status Scale (EDSS) ≥8.0 [19]
3. Primary or secondary progressive course
4. Presence of a carer (family member, relative, or friend, who is next of kin or is key decision maker as designated by the (cognitively competent) MS patient and with whom the patient shares his/her life)
5. At least two unmet care needs among the categories identified in the PeNSAMI Phase 1 qualitative study (Table 1) [11], or the patient declares for comfort care only

**Table 1:** **List of main care need categories, as identified in the PeNSAMI Phase 1 qualitative study** [11]

**Domain**	**Category**
**‘** ***Managing everyday life*** **’**	Symptoms management
	Personal care/hygiene
	Activities of daily living
	Outdoor mobility and transport
**‘** ***Psychosocial*** **’**	Relationships/communication
	Leisure/holidays
	Psychological well-being/social role
**‘** ***Organization*** **’**	Information
	Access to services
	Co-ordination of services
	Competent professionals
6. One or more of the following: significant complex symptoms/medical complications, dysphagia/poor nutritional status, communication difficulties [20].

Exclusion criteria are:

1. Hospitalized/institutionalized patients
2. Patients already receiving palliative care
3. Dyads living out of study area

#### Recruitment

Starting on 10 January 2015 participants are being recruited from the three participating centers. Recruitment closes at the end of June 2015.

#### Informed consent and pre-study evaluation (Visit 0)

Potentially eligible patients are identified by the center principal investigator (PI) by consulting the MS center database and by contacts with general practitioners (GPs), and other health personnel involved in the care of severely affected MS patients. MS patient and their carer are approached by a HPA team member to give informed consent to participate. If consent is obtained, baseline electronic case record forms (eCRFs) are completed.

#### Baseline visit (Visit 1)

After pre-study evaluation, participants identified as meeting the eligibility criteria are assessed at home by the blind examiner who confirms the inclusion criteria, administers the outcome instruments and initiates the randomization procedure. A card showing the time and date of the three- and six-month follow-up visits is given to the dyad.

#### Randomization

Eligible study participants are randomized to either HPA or UC using third-party, web-based computerized randomization software. A stratified minimization algorithm is used to ensure balance of possible prognostic factors across the two groups (EDSS (8.0 to 8.5, 9.0 to 9.5), presence of severe cognitive compromise (clinical judgment), and center (Milan, Rome, Catania)). Following randomization, an email informing on dyad assignment is sent to the PI and the HPA team. The team informs the dyad about assignment. The team also contacts the patient’s caring physician (GP, neurologist or other physician responsible for the patient’s care) to inform him/her about the study and (for dyads assigned to HPA) define a common agenda.

#### Blinding

To reduce measurement bias, the baseline and follow-up assessments are performed by independent examiners blind to treatment assignment (one examiner plus backup at each center, both trained, neither involved in HPA delivery). Prior to each follow-up visit, study dyads will be reminded not to disclose their allocation by mentioning any contact with the HPA team to the blind examiner. The examiner will be asked to guess dyad status (HPA vs. UC) after the three- and six-month visits to assess whether blinding is maintained.

#### HPA group

The trial intervention is based on the principles of palliative care as described in the 2010 guidance document of the UK National Council for Palliative Care, Neurological Alliance, and National End of Life Care Programme [21]. Each center has a HPA team consisting of four professionals: a physician (neurologist, physiatrist or palliative specialist), nurse, psychologist, and social worker. The HPA team nurse is the case manager and team leader: the nurses of the centers of Milan and Rome have degrees in palliative care; the Catania nurse attended a FARO Foundation week-long individual training course in September 2014. All HPA team members were trained in the HPA intervention at the FARO Foundation, Turin, 20 to 21 February 2014. HPA teams will meet about two months after trial initiation (more often if necessary) to share experiences and fine-tune the protocol.

Each HPA team meets regularly to discuss the management of dyads in their charge. Initially, the team forms a comprehensive assessment of each dyad based on information provided by the blind examiner visit (available to the HPA team via the eCRF and the web-based information system) and that provided by the team home visit. The HPA content for each dyad is then developed, discussed with the caring physician, and delivered over six months, with involvement of local services. The intervention is not intended to replace existing services but to complement and enhance them, minimizing duplication of effort. Subsequently, the HPA team verifies program implementation, and reviews and modifies it as necessary.

Home visits by one or more team members will take place at least twice a month in the first trimester, and as needed thereafter. Which professionals attend for home visits depends on the intensity of care and the type of symptoms (for example physician for pain management, nurse for bed sore treatment). A dedicated phone number is given to dyads to enable easy communication with the HPA team. The team is not on call for patients: in the event of emergencies, dyads contact the caring physician or emergency medical services.

We expect HRQOL and other study outcomes to improve in the first trimester, so thereafter frequent home visits could be useless or intrusive [15]. However, if symptoms and HRQOL worsen, or end of life issues manifest, intensity of care will be maintained or increased. All HPA team activities/interventions will be recorded in the patient study record, kept at the patient’s home and available to all health professionals/caregivers. To preserve blinding, before visits 2 and 3, dyads will be reminded to keep the patient study record out of the blind examiner’s sight (for example by placing it in a drawer).

#### UC group

UC consists of the health and social services normally provided by the Italian National Health Service in the study area (for example by GP, district nursing service, social services, neurologists and other specialists, and emergency services). Patient-carer dyads assigned to UC receive the three blind examiner visits and the monthly telephone interviews, but not the HPA team visits (except Visit 0). At the end of the study, dyads who received UC will be offered the HPA.

#### Primary outcomes

Primary outcomes are changes in patient HRQOL (Schedule for the Evaluation of Individual Quality of Life-Direct Weighting, SEIQoL-DW) and MS symptoms (Palliative care Outcome Scale-Symptoms-MS, POS-S-MS). The SEIQoL-DW is a brief instrument, derived from the schedule for evaluation of individual quality of life (SEIQoL). It allows respondents to nominate areas of life that are most important in determining their HRQOL. The level of satisfaction/functioning in each of these areas is then recorded [22-26]. The SEIQoL-DW index is obtained from the satisfaction with and weighting for each area, and ranges from 0 (worst possible score) to 100 (best possible score). SEIQoL-DW has been used in neurological diseases [27-30], and was the primary outcome measure of the Ne-Pal trial [15].

POS-S-MS (primary outcome measure) and the Core-Palliative care Outcome Scale (Core-POS) were developed and validated for use with palliative care patients to assess emotional, psychological and spiritual needs, and provision of information and support [31,32]. Core-POS has been used in patients with advanced cancer and other conditions including dementia, motor neuron disease, and MS [33,34]. It consists of 10 items, which are scored from 0 (best) to 4 (worst). The score is the sum of the scores from each question, and therefore can range from 0 to 40. There are patient, carer, and staff versions, each requiring about 10 minutes to complete [31,35]. Generally, the patient is asked about needs over the preceding three days, but other time frames have been used [36]: in this study, we asked about the preceding seven days.

POS-symptoms (POS-S) is a scale enquiring about 10 symptoms with two open questions (which symptom affected the patient the most; which improved most). It was developed for use in cancer patients and is used alongside the Core-POS for assessing common physical symptoms. Disease-specific versions of POS-S have been developed for end-stage kidney disease, Parkinson’s disease, and MS [33]. POS-S-MS comprises 18 items relating to MS symptoms, plus an open question (0 to 4 scale) over the preceding seven days [34].

The psychometric properties of Core-POS and POS-S-MS have been assessed in patients with severe MS: both are acceptable, reliable, and valid in this population [34].

In the present RCT, MS patients with severe cognitive impairment do not complete the questionnaires: the caregiver versions of Core-POS and POS-S-MS are used.

#### Secondary outcomes

Secondary outcome measures are HRQOL as assessed by Core-POS [34] and European Quality of Life Five Dimensions (EQ-5D) [37,38]; mood as assessed by the Hospital Anxiety and Depression Scale (HADS) [39,40]; impairment/activity limitations as assessed by EDSS [19] and Functional Independence Measure (FIM) [41,42]; and direct and indirect tangible costs are assessed by the MS foundation Costs Questionnaire (MSCQ) [43].

Carer outcomes are: HRQOL as measured by Short Form 36 (SF-36) [44,45] and EQ-5D; mood as measured by the HADS; and carer burden as measured by the Zarit Burden Interview (ZBI) [46,47].

Recruitment rates, reasons for exclusion, time to delivery of interventions, adherence to intervention (see the HPA group), protocol deviations, incorporation with standard care, unplanned hospital admissions, referrals to hospice, deaths (place and cause) are also considered over the six-month period.

Outcome assessments (Table 2) are carried out at baseline, and after three and six months, by a blind examiner at the patient’s home. In addition, six telephone interviews are performed, on a monthly basis, by a trained interviewer who administers the full MSCQ (at three and six months) and pertinent MSCQ sections (at one, two, four and five months).

**Table 2:** **Outcome measures and timing of follow-up**

	**Baseline**	**Three-month follow-up**	**Six-month follow-up**
General characteristics	X	X	X
MS history	X	X	X
Medical history	X	X	X
EDSS	X	X	X
FIM	X	X	X
SEIQoL-DW^*^	X	X	X
Core-POS^**^	X	X	X
POS-S-MS^**^	X	X	X
HADS^*^	X	X	X
EQ-5D^*^	X	X	X
MSCQ	X	†	†
Carer characteristics /medical history	X	X	X
Carer HADS	X	X	X
Carer SF-36	X	X	X
Carer EQ-5D	X	X	X
Carer ZBI	X	X	X

To preserve blinding, all outcome measures (except MSCQ) are assessed during examiner visits at three and six months follow-up. ^*^Not assessed in MS patients with severe cognitive compromise or unable to communicate; ^**^carer versions used in MS patients with severe cognitive compromise or unable to communicate; ^†^assessed by phone interview. Core-POS, Core-Palliative care Outcome Scale; EDSS, Expanded Disability Status Scale; EQ-5D, European Quality of Life Five Dimensions; FIM, Functional Independence Measure; HADS, Hospital Anxiety and Depression Scale; MSCQ, Multiple Sclerosis foundation Costs Questionnaire; MS, multiple sclerosis; POS-S-MS, Palliative care Outcome Scale-Symptoms-Multiple Sclerosis; SF-36, Short Form 36; SEIQoL-DW, Schedule for the Evaluation of Individual Quality of Life-Direct Weighting; ZBI, Zarit Burden Interview.

#### Safety

We expect no serious psychological, physical, social, or legal risks to derive from exposure to the study intervention. We cannot exclude, however, that the study intervention will have a negative effect on patient status. The following serious adverse events (SAEs) are being monitored:

- Emergency room visits that do not result in hospital admission
- Hospitalizations (overnight stay at hospital or emergency room for observation or treatment)
- Death (any cause)

Hospitalization for elective surgery, routine or planned clinical procedures are not considered SAEs.

#### Withdrawals and loss to follow-up

Dyads can withdraw from the study at any time. The ‘intention-to-treat’ principle will apply for participants who withdraw or are lost to follow-up.

#### Independent data and safety monitoring committee (IDSMC)

The IDSMC monitors SAE reports throughout the trial, and oversees trial progress, ensuring that it is conducted, recorded, and reported in accordance with the protocol, good clinical practice, and applicable regulatory requirements. The IDSMC provides recommendations about stopping or continuing the trial in the event of harm, undue risks, or futility. IDSMC members (Appendix 1) met (via teleconference) prior to the start of enrolment. Subsequent IDMSC teleconferences are scheduled every four months, and at trial termination, or more frequently as necessary.

#### Sample size

The sample size was based on previous data for SEIQoL-DW [15] and POS-S-MS [8]; for both outcomes we considered changes at three months compared to baseline.

SEIQoL-DW: a sample size of 21 patients assigned to HPA (study intervention) and 11 assigned to UC (control) has a power of 80% to detect an assumed mean change of score of 31.5 (standard deviation (SD) 12.8) in the HPA group compared to a change of 12.1 (SD 19.3) (null hypothesis) in the UC group, at alpha level 0.05 using a two-sided, two-sample *t* test. Assuming 20% dropout, 25 patients are required in the HPA group and 13 in the UC group (total sample size 38).

POS-S-MS: a sample size of 41 HPA and 21 UC has a power of 85% to detect an assumed mean change of −0.4 (SD 0.5) in the HPA group, with a concomitant change of 0.2 (SD 0.8) (null hypothesis) in the UC group; assuming an alpha level of 0.05 and a two-sided, two-sample *t* test. Assuming 20% dropout, 49 patients are required in the HPA group and 25 patients in the UC group (total sample size 74).

It is expected that up to 50% of MS patients have severe cognitive compromise, and will not be able to complete SEIQoL-DW, in these the only primary endpoint will be the POS-S-MS. We aim to recruit 50 patients in the HPA group and 25 patients in the UC group, thereby achieving an exact 2:1 ratio of intervention to control.

#### Statistical analyses

Continuous data will be summarized using means, medians, SDs, minimums and maximums. Between-group comparisons will employ either the two-sided unpaired *t* test or Wilcoxon’s two-sided two-sample test for non-normal data. Distributions will be tested for normality using the Shapiro-Wilk test. Correlations will be estimated using Spearman’s or Pearson’s coefficients. Categorical data will be compared by χ2 or Fisher’s exact test.

Longitudinal changes in SEIQoL-DW and POS-S-MS will be analyzed using linear mixed models for longitudinal data [48,49]. All tests will be two-tailed. *P* values <0.05 will be considered significant. For the primary intention-to-treat analyses, multiple imputation of missing values will employ Rubin’s approach. The analyses will be performed with Stata Statistical Software, release 12 (StataCorp LP, College Station, TX, USA), or SAS, release 9.2 (SAS Institute Inc., Cary, NC, USA).

### Nested qualitative study

The qualitative study consists of two parts: personal, semi-structured interview with MS patients and carers, and focus group meetings (FGMs) of patients’ caring physicians. The aim is to evaluate the experience of participants in order to gain insights into the strengths and weaknesses of the HPA.

#### Informed consent

Prior to participation, potentially eligible qualitative study participants (patients and carers who received the HPA and caring physicians of patients) will be approached by the center PI or a HPA team member, and asked to provide informed consent to participate.

#### Personal semi-structured interviews

We will use a purposive sampling technique to identify consenting HPA dyads in whom primary outcomes (SEIQoL-DW and POS-S-MS) were either among the highest or lowest of the distribution, at the three- or six-month follow-ups. For the dyads thus identified, patients and carers will be interviewed separately, within six months of trial completion. Interviews will be face-to-face, conducted by a trained psychologist in the patient’s home, last for a maximum of 60 minutes (patients) or 90 minutes (caregivers), and will be audio-recorded and transcribed in full. Interviewees will be asked about the acceptability and utility of the HPA intervention as a whole and other issues that arise or seem pertinent (for example relation with professionals, timing, integration with current care). The interviewer will also note participant behavior and other potentially useful non-verbal aspects that emerge. Only carers will be interviewed if the patient has severe cognitive compromise. A minimum of 12 dyads (four at each center) will be included, with sampling continuing until no new themes emerge from the data (data saturation) [50].

#### Focus group meetings

Three FGMs will be conducted (one at each center), their objective is to reveal physicians’ experiences and views about the study intervention. Positive and negative aspects will be solicited. All caring physicians of patients who received the HPA will be invited to participate. Each FGM will be audio-recorded, has a moderator (a trained psychologist) and co-moderator, and an expected minimum of five physician participants.

#### Analysis

The methods of framework analysis [50-52] will be applied to the data. Framework analysis uses a systematic approach to analyzing the content of interviews and FGMs, to thereby identify themes and categories of responses. The interviewers/moderators will review the audio recordings and written notes, and produce interview/meeting transcripts. Interviews and FGMs will be analyzed in successive steps, each corresponding to an increasing level of generalization [53]. Two researchers will analyze the interviews and FGMs, first independently and then jointly. Each FGM report will be submitted to FGM participants for review (respondent validation). The interview and FGM reports will then be considered jointly to produce a more comprehensive analysis of the data (triangulation) [54].

### Study organization

The study investigators/committees are reported in Appendix 1. The trial steering committee is the main decision-making body. It consists of local PIs, patient and consumer representatives, and experts in MS, palliative care, rehabilitation, and methodology. The steering committee meets at least once a year and is the study writing committee (on behalf of all investigators).

The IDSMC is chaired by a palliative physician, and includes a neurologist and methodologist. The IDSMC met (via teleconference) before starting accrual; subsequent teleconferences are scheduled every four months, and at trial termination. IDSMC reports are sent to the trial steering committee.

The qualitative study panel is responsible for planning, conducting and analyzing the qualitative study. It consists of two psychologists with expertise in qualitative research, and the psychologists who will conduct interviews and FGMs.

Trial data are entered into a web-based, password-protected data management system/eCRF that permits edit-audit trails (by authorized trained personnel). Paper copies of eCRFs and any (anonymized) supporting documentation is stored securely at participating centers, with identifying contact details and signed consent forms stored separately, for seven years.

## Discussion

PeNSAMI is a RCT with a nested qualitative study designed to test the effectiveness of a HPA on people with severe MS and their carers. This trial will primarily assess the effect of the HPA on patient HRQOL and symptoms; secondarily it will assess patient functional status and mood; carer HRQOL, mood and caregiving burden; costs, incorporation with standard care, unplanned hospital admissions, referrals to hospice, and deaths over the six-month period.

To our knowledge, this is the first clinical trial of a palliative approach to people with severe MS that adopts a single-blind (examiner) and multicenter design. By also evaluating the experiences of the key players involved in the HPA, we will better identify intervention strengths and limitations. Integration of these qualitative findings with the quantitative findings will maximize possibilities for improving the HPA [55]. Additionally, careful documentation of the content, processes and outcomes of the intervention will make possible its replication and wide implementation [55].

We decided on a randomized controlled study because of its potential to produce unbiased data on intervention efficacy. UC was considered the most suitable comparator, since a sham intervention was not feasible. Note, however, that regular outcome assessment with frequent contacts ensures that the control group receives a considerable amount of attention. For ethical reasons and to encourage participation, we decided to assign eligible dyads to HPA vs. UC in a 2:1 ratio, and also to offer control dyads the HPA at the end of follow-up.

### Expected benefits

Fragmentation of care across different health care sectors is a recognized problem, particularly for patients with complex care needs and co-morbidities. The HPA is not intended to replace existing services but to complement, collaborate with, and enhance them, minimizing duplication of effort. The potential for HPA integration into existing services is a strength of our trial.

Health professionals caring for people with severe MS themselves have several unmet needs and feel overstretched in their daily work. Physicians express feelings of helplessness or anger in the face of cuts in funding, and having to work under time pressure and in complex social situations [11]. They also feel hampered by inadequate communication skills (not taught at university) [10,56].

In addition to providing valuable data, the trial affords interdisciplinary training for the health professionals involved. By making palliative care professionals work with MS neurology and rehabilitation professionals [57], the trial should encourage greater awareness and more effective management of people with severe MS.

Effective exchange between the dyad, the caring physician and the HPA team should result in treatment decisions in the best interests of MS patients and the avoidance of inappropriate hospital admissions. It is also expected that the trial will empower people with severe MS (and their carers) by providing them with information and education on their condition, encouraging them to be active participants in care and decision making, including advance health directives where appropriate.

### Limitations and concerns

We estimated a sample size of 75 patients, which is rather small for a phase II/III trial. We based our sample size estimate on published data: for POS-S-MS, these data come from MS patients with similar characteristics to our study population [8], and for SEIQoL-DW, from people with severe MS or other neurodegenerative conditions [15]. In view of our limited sample size, we used adaptive (minimization) randomization to ensure comparability of study arms.

Another limitation is that, in contrast with previous trials [8,15], our trial did not originate from palliative services, which in theory can directly address needs identified. Our HPA aims to activate existing services or bring them to the attention of the dyad. The extent to which these aims are achieved cannot be predicted.

Finally, having a blind examiner renders trial procedures considerably more complex: the blind examiner must have no contact with the HPA team; dyads must be briefed about the importance of keeping the examiner blinded; and, prior to three- and six-month visits, the blind examiner is instructed to caution dyads against disclosing their assignment or mentioning issues related to study procedures. In addition, costs and intensity of care outcomes, as assessed by the MSCQ, are assessed by telephone interviews at three and six months conducted by an independent professional - again to prevent examiner unmasking.

## Trial status

Patient recruitment started in January 2015.

## Appendix 1

PeNSAMI project investigators.

Steering Committee: R Amadeo, A Giordano, M Ponzio, MG Grasso, A Lugaresi, F Patti, G Martino, L Palmisano, S Veronese, P Zaratin, MA Battaglia, A Solari.

Data Management and Analysis Committee: A Giordano, D Radice (statistician): *Division of Epidemiology and Biostatistics*, *European Institute of Oncology*, *Milan*, M Ponzio (statistician), G Ferrari, A Solari.

Independent Data and Safety Monitoring Committee: DJ Oliver: *Wisdom Hospice*, *University of Kent, Rochester*, *Kent, UK*; E Pucci: *Neurology Unit*, *Ospedale Provinciale di Macerata*, *Macerata*; L Tesio: *Department of Biomedical Sciences for Health*, *University of Milan*, *Milan.*

Qualitative Analysis Panel: E Bianchi, E Pietrolongo, A Solari, A Giordano, I Rossi, S Cilia, M Giuntoli, C Borreani.

Literature Review Panel: MG Grasso, L Palmisano, A Fittipaldo, A Giordano.

Intervention Panel: C Cugno, R Causarano, P Morino: *‘Ex Convento delle Oblate’ Hospice, Local Health Unit of Florence, Florence*, S Veronese.

Centers and Investigators: *AISM Liguria Region Rehabilitation Service*, *Genoa*: ML Lopes de Carvalho, M Giuntoli, R Motta, MA Battaglia; *Antea Charitable Association*, *Rome*: G Casale, MC Stefanelli; *FARO Charitable Foundation*, *Turin*: S Veronese, C Cugno; *Foundation IRCCS Istituto Nazionale per la Cura dei Tumori*, *Milan*: C Borreani, E Bianchi; *Foundation IRCCS Neurological Institute C Besta, Milan*: A Solari, P Confalonieri, A Giovannetti, V Torri Clerici, E Rossetti, A Totis, A Campanella, A Giordano, F Martini, A Fittipaldo, G Ferrari, R Mantegazza; *Foundation IRCCS S Lucia Rehabilitation Hospital*, *Rome*: MG Grasso, I Rossi, E Troisi, A Pompa, L Tucci, F Ippoliti, G Morone, A Fusco; *Istituto Superiore di Sanità*, *Rome*: L Palmisano; *Associazione Italiana Sclerosi Multipla (AISM)*, *Genoa*: R Amadeo, G Martino; *Fondazione Italiana Sclerosi Multipla (FISM)*, *Genoa*: P Zaratin, M Ponzio, MA Battaglia; *Niguarda Ca’ Granda Hospital*, *Milan*: R Causarano, D Da Col, B Lissoni; *G d’Annunzio University*, *Chieti-Pescara*, *Chieti*: A Lugaresi, E Pietrolongo, M Onofrj; *University Hospital Policlinico Vittorio Emanuele*, *Catania*: F Patti, S Cilia, C Leone, V Cascio, V Cimino, G Occhipinti, A Pappalardo, C Cavallaro, F Zagari.

## Abbreviations

Core-POS: Core-Palliative care Outcome Scale
eCRF: electronic case record form
EDSS: Expanded Disability Status Scale
EQ-5D: European Quality of Life Five Dimensions
FGM: focus group meeting
FIM: Functional Independence Measure
GP: general practitioner
HADS: Hospital Anxiety and Depression Scale
HPA: home-based palliative approach
HRQOL: health-related quality of life
IDSMC: Independent Data and Safety Monitoring Committee
MSCQ: Multiple Sclerosis foundation Costs Questionnaire
MS: multiple sclerosis
PI: principal investigator
POS-S-MS: Palliative care Outcome Scale-Symptoms-Multiple Sclerosis
RCT: randomized controlled trial
SAE: serious adverse event
SD: standard deviation
SF-36: Short Form 36
SEIQoL-DW: Schedule for the Evaluation of Individual Quality of Life - Direct Weighting
UC: usual care
ZBI: Zarit Burden Interview
