# Erratum to: ‘Home-based palliative approach for people with severe multiple sclerosis and their carers: study protocol for a randomized controlled trial’

**DOI:** 10.1186/s13063-016-1228-1

**Published:** 2016-02-16

**Authors:** A. Solari, A. Giordano, M. G. Grasso, P. Confalonieri, F. Patti, A. Lugaresi, L. Palmisano, R. Amadeo, G. Martino, M. Ponzio, G. Casale, C. Borreani, R. Causarano, S. Veronese, P. Zaratin, M. A. Battaglia

**Affiliations:** Unit of Neuroepidemiology, Foundation IRCCS Neurological Institute C. Besta, Milan, Italy; Multiple Sclerosis Unit, Foundation IRCCS S. Lucia Rehabilitation Hospital, Rome, Italy; Department of Medical and Surgical Sciences and Advanced Technologies, University of Catania; MS Center, Neurology Clinic, University Hospital Policlinico Vittorio Emanuele, Catania, Italy; Department of Neuroscience, Imaging and Clinical Sciences, G. d’Annunzio University of Chieti-Pescara, Chieti, Italy; Department of Therapeutic Research and Medicine Evaluation, Istituto Superiore di Sanità, Rome, Italy; Associazione Italiana Sclerosi Multipla, Genoa, Italy; Fondazione Italiana Sclerosi Multipla, Genoa, Italy; Antea Charitable Association, Rome, Italy; Unit of Clinical Psychology, Foundation IRCCS Istituto Nazionale per la Cura dei Tumori, Milan, Italy; Unit of Palliative Care-Hospice, Niguarda Ca’ Granda Hospital, Milan, Italy; FARO Charitable Foundation, Turin, Italy

Unfortunately, the original version of this article [1] contained an error. The affiliation section for Luigi Tesio in Appendix 1 of this article was included incorrectly as it was missing a second affiliation. The correct affiliations for Luigi Tesio can be found below.

1. Department of Biomedical Sciences for Health, Università degli Studi,
2. The Istituto Auxologico Italiano, IRCCS, Milan, Italy
