# Erratum to: Home-based palliative approach for people with severe multiple sclerosis and their carers: study protocol for a randomized controlled trial

**DOI:** 10.1186/s13063-017-1787-9

**Published:** 2017-01-23

**Authors:** A. Solari

**Affiliations:** Unit of Neuroepidemiology, Foundation IRCCS Neurological Institute C. Besta, Via Celoria 11, 20133 Milan, Italy

## Erratum

Unfortunately, the original version of this article [1] contained an error. The correct paragraph can be found below:

SEIQoL-DW: a sample size of 21 patients assigned to HPA (study intervention) and 11 assigned to UC (control) has a power of 80% to detect an assumed mean change of score of 12.1 (standard deviation (SD) 12.8) in the HPA group compared to a change of -7.4 (SD 19.3) (null hypothesis) in the UC group, at alpha level 0.05 using a two-sided, two-sample t test. Assuming 20% dropout, 25 patients are required in the HPA group and 13 in the UC group (total sample size 38).
